# Development of open access tool for automatic use factor calculation using DICOM-RT patient data

**DOI:** 10.1007/s13246-023-01272-1

**Published:** 2023-07-20

**Authors:** Dong Hyeok Choi, Dong Wook Kim, So Hyun Park, So Hyun Ahn, Woo Sang Ahn, Rena Lee, Jin Sung Kim

**Affiliations:** 1 Department of Medicine, Yonsei University College of Medicine, Seoul, Korea; 2 Medical Physics and Biomedical Engineering Lab (MPBEL), Yonsei University College of Medicine, Seoul, South Korea; 3 Department of Radiation Oncology, Heavy Ion Therapy Research Institute, Yonsei Cancer Center, Yonsei University College of Medicine, Seoul, South Korea; 4 Department of Radiation Oncology, Jeju National University Hospital, Jeju University College of Medicine, Jeju, Republic of Korea; 5 Ewha Medical Research Institute, School of Medicine, Ewha Womans University, Seoul, Republic of Korea; 6 Department of Radiation Oncology, Gangneung Asan Hospital, University of Ulsan College of Medicine, Gangneung, Republic of Korea; 7 Department of Biomedical Engineering, School of Medicine, Ewha Womans University, Seoul, South Korea

**Keywords:** Use factor, DICOM-RT, Monitor unit, LINAC, NCRP 151, Shielding evaluation

## Abstract

Our study recalculated the use factor of linear accelerators (LINACs) by using an in-house program based on Digital Imaging and Communications in Medicine radiation therapy (DICOM-RT). We considered the impact of advancements and changes in treatment trends, including modality, technology, and radiation dose, on the use factor, which is one of the shielding parameters. In accordance with the methodology described in the NCRP 151 report, we computed the use factor for four linear accelerators (LINACs) across three hospitals. We analyzed the results based on the treatment techniques and treatment sites for three-dimensional conformal radiation therapy (3D-CRT) and intensity modulated radiation therapy or volumetric modulated arc therapy. Our findings revealed that the use factors obtained at 45° and 90° were 14.8% and 13.5% higher than those of the NCRP 151 report. In treatment rooms with a high 3D-CRT ratio, the use factor at a specific angle differed by up to 14.6% relative to the NCRP 151 report value. Our results showed a large difference in the use factor for specific sites such as the breast and spine, so it is recommended that each institution recalculate the use factor using patient’s data.

**Supplementary Information:**

The online version contains supplementary material available at 10.1007/s13246-023-01272-1.

## Introduction

The design of a radiation shielding system for a linear accelerator (LINAC) facility requires consideration of various factors, including workload, use factor, and occupancy factor, which are important in radiation oncology. These factors are affected by the treatment techniques, radiation energy, number of patients, and administered dose [1, 2]. The National Council on Radiation Protection and Measurements (NCRP) has updated its guidelines and recommendations for radiation shielding in recent years, taking into account the latest treatment modalities, techniques, and doses, as well as current trends in radiation therapy [3–8]. From 1976 to 2004, the radiation shielding guidelines and recommendations documented in NCRP 49 were mainly considered for medical X-ray imaging facilities [9, 10].

The use factor is defined as the ratio of the beam used according to the angle of the gantry in the radiation therapy equipment. For example, if beams of 200 MU and 300 MU are delivered at gantry angles of 0 and 90 degrees, respectively, the use factors will be 0.4/0.6/0.0/0.0 for 0/90/180/270 degrees, respectively. In this example, the ratio of the use factor in the direction of the gantry angle of 90 degrees is high. It means that the amount of radiation delivered to the radiation protection wall in that direction is high. Therefore, a higher level of shielding design is required.

In the past, NCRP 26 suggested a use factor of 1/4 for each of the four directions at 90° for all walls used as primary barriers in diagnostic X-ray facilities [3]. However, NCRP 151 now provides up-to-date standard guidelines for radiation shielding design of most radiation oncology equipment. These new guidelines take into account various techniques, usage rates, and use factors, including gantry angle intervals, intensity modulated radiation therapy (IMRT) “efficiency” factor, scatter fraction, and particle production [7, 8].

The use factor for each gantry angle plays a crucial role in radiation shielding calculations, and it is highly dependent on the treatment technique used [11]. In fact, a study conducted at Memorial Sloan–Kettering Cancer Center (MSKCC) revealed variations in the use factors depending on the treatment techniques. This study analyzed the data of patients treated with a total of 16 treatment devices from 2006 to 2015, and it found that the use factors changed due to the alterations in treatment techniques and workload [12].

Over the past few years, advanced radiotherapy techniques such as IMRT and volumetric modulated arc therapy (VMAT) have become increasingly popular due to their ability to provide better target coverage and limit exposure to adjacent healthy organs [13–20]. In contrast to conventional treatment techniques, IMRT and VMAT techniques use approximately 2–10 times the MU values [21–25]. As a result, the amount of MU reaching the treatment room barrier is influenced by the gantry angle, and the use factor calculated as a proportion for the entire gantry angle may be affected by changes in treatment techniques [26]. Consequently, it is essential to reevaluate the use factor in radiation shielding design to account for the growing use of these advanced techniques [12, 13, 27].

In this study, we used an in-house calculation program based on DICOM-RT, which takes into account the monitor unit (MU), to reanalyze the use factor for radiation shielding according to treatment sites and institutions. This was done to incorporate the impact of recent developments and changes in treatment trends.

## Methods and materials

### Data acquisition

This study collected Digital Imaging and Communications in Medicine radiation therapy (DICOM-RT) plan files from a total of 2,811 patients who received radiation therapy from January to December 2020 from the radiation oncology departments of Yonsei Cancer Center (YCC), Gangneung Asan Hospital (GNAH), and Jeju National University Hospital (JNUH). The patient identification information was anonymized using MATLAB or a treatment planning system, and each institution obtained Institutional Review Board approval before collecting patient data data (YCC 4-2021-0857, GNAH 2021-04-034, and JNUH 2022-01-006). The LINAC models, beam energies, and treatment techniques for each hospital are listed in Table 1, and the treatment rooms for YCC, GNAH, and JNUH are named Room A, Room B, and Room C, respectively.

**Table 1:** Summary of treatment machines, beam energies, and treatment techniques for 1 year (January 01, 2020, to December 31, 2020)

Vault #	Model (Manufacturer)	Photons (MV)	Electrons (MeV)	Treatment techniques
A-1	Infinity (Elekta)	6, 10	6, 9, 12, 16, 20	3D-CRT, VMAT
A-2	Infinity (Elekta)	4, 6, 10	6, 9, 12, 15, 18	3D-CRT, VMAT
B	TrueBeam (Varian)	6, 10, 15, 6FFF, 10FFF	6, 9, 12, 16, 20	3D-CRT, IMRT, VMAT, SBRT, SRS
C	Clinac iX (Varian)	6, 15	6, 9, 12, 16, 20	3D-CRT, IMRT, VMAT

### DATA analysis

**Fig. 1:**
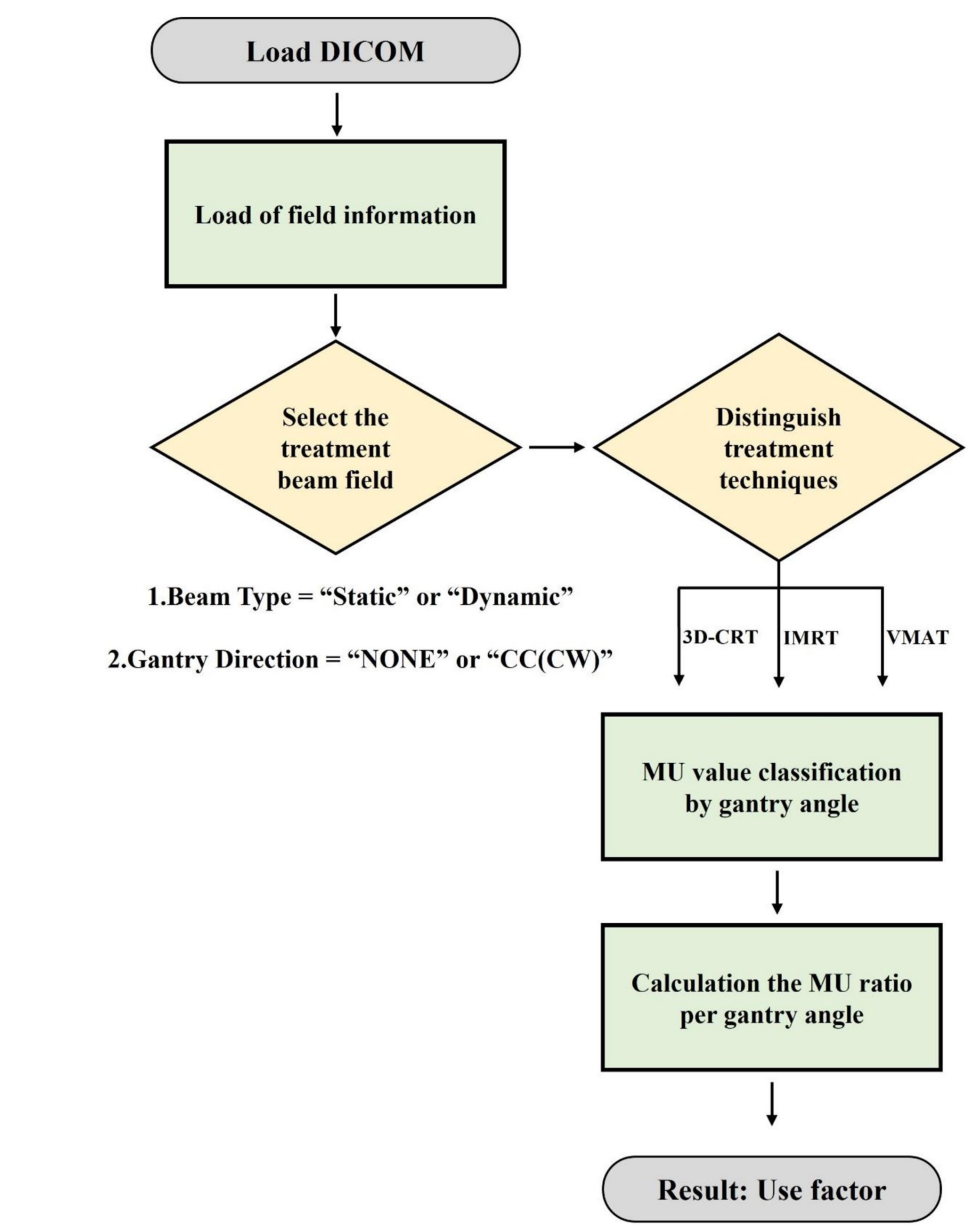
Flowchart for obtaining use factor from the DICOM-RT plan file

To analyze the use factors, a program was created using MATLAB (MathWorks Inc.). This program uses information from the patients’ DICOM-RT plan, such as the treatment technique, monitor unit, and gantry angle. The program uses three types of gantry angle intervals: 90°, 45°, 30°, and 10°. Each angle interval covers half of the specific angle (for example, 45–135° for 90°). The flow chart of the program is depicted in Fig. 1, and the detailed explanation of the program code is as follows.

1. DICOM-RT plan file is used as input for user factor analysis.
2. If the Beam dose value is present in the information of the DICOM-RT plan file, the beam is considered a treatment beam.
3. The Beam Type information is used to distinguish between 3D-CRT and IMRT & VMAT treatment techniques. If the Beam Type is Static, it is 3D-CRT, and if it is Dynamic, it is either IMRT or VMAT treatment technique.
4. The MU values for each gantry angle are stored in the Control Point Sequence of the selected treatment beams, and the MU values are classified by gantry angle for each patient using this information.
5. The classified MU values are calculated as a ratio for a specific angle (90°, 45°, 30°, and 10°), regardless of the treatment technique. The process of classifying MU values is performed without distinguishing between treatment techniques.
6. The use factor is outputted for each treatment technique and for a specific angle based on the classified MU values.

### Evaluation

To evaluate the impact of treatment technique and treatment site on the use factor, the study analyzed actual patient data from three institutions over the course of one year and compared the results to the use factors suggested in the NCRP 151 report. The treatment techniques examined were 3D-CRT, IMRT, and VMAT, and the treatment sites included breast, head and neck (H&N), abdomen, chest, pelvis, spine, and extremity.

## Results

Table 2 presents the number of patients, treatment sites, and treatment techniques for each institution. Among the four treatment rooms, Room C had the highest percentage of 3D-CRT treatment, accounting for 34.1 ± 6.0% of the total treatment. The LINAC in Room B had the highest IMRT ratio, accounting for 60.0 ± 5.9% of the total treatment. Room A-1 and Room A-2 had the highest VMAT ratio, accounting for 92.0 ± 7.4% and 81.8 ± 7.9% of the total treatment, respectively.

**Table 2:** Treatment information for each vault

Item	A-1		A-2		B		C	
	Sum	Statistics (%)	Sum	Statistics (%)	Sum	Statistics (%)	Sum	Statistics (%)
Number of fractions	3401		7051		20,402		10,733	
Number of new patients	433		885		817		676	
Number of treatment sites	45		79		74		54	
IMRT	0	0	0	0	4786	44.9	889	8.3
VMAT	3119	91.7	5774	81.9	4645	43.6	6315	59.2
3D	282	8.3	1277	18.1	1110	10.4	3736	35.1
SBRT	0	0	0	0	118	1.1	0	0

### Use factors for different treatment techniques

Table 3 compares the use factors recommended by NCRP 151 with those calculated from patient data. The comparison is shown for 90° and 45° intervals. While the total use factor for each institution shows different tendencies depending on the ratio of techniques for the 90° intervals, the use factor analysis at each 45° interval confirms that the use factors for diagonal directions (45°, 135°, 225°, and 315°) differ by up to 14.8% (Room C, 180º) compared to the NCRP 151 values. At the standard angles of 0°, 90°, 180°, and 270°, the use factors differ from those reported in NCRP 151 by up to 13.5% (Room C, 90º).

**Table 3:** Use factors calculated from the patient data and those documented in NCRP 151 according to the treatment technique. (Unit: %)

	Interval	90º				45º							
	Angle	0º	90º	180º	270º	0º	45º	90º	135º	180º	225º	270º	315º
Institution	**NCRP 151**	**31.0**	**21.3**	**26.3**	**21.3**	**25.6**	**5.8**	**15.9**	**4.0**	**23.0**	**4.0**	**15.9**	**5.8**
A-1	3D-CRT	17.1	21.2	28.5	33.2	12.0	4.1	17.1	14.3	14.2	15.2	18.0	5.1
	IMRT	-	-	-	-	-	-	-	-	-	-	-	-
	VMAT	25.9	24.8	22.5	26.8	13.5	12.7	12.2	11.8	10.7	13.3	13.5	12.4
	Total	25.1	24.7	23.6	26.6	13.2	11.9	12.8	12.2	11.4	13.2	13.4	11.9
A-2	3D-CRT	33.4	18.9	27.4	20.3	20.6	7.6	11.3	15.8	11.6	9.4	11.0	12.8
	IMRT	-	-	-	-	-	-	-	-	-	-	-	-
	VMAT	25.9	25.6	19.4	29.1	13.1	14.0	11.6	9.8	9.6	13.6	15.5	12.8
	Total	26.3	24.8	20.4	28.5	13.4	13.5	11.3	10.6	9.8	13.4	15.1	12.9
B	3D-CRT	32.2	16.6	31.2	20.0	20.6	10.5	6.1	13.8	17.4	13.6	6.4	11.6
	IMRT	28.9	25.3	21.6	24.2	13.6	19.1	6.2	11.4	10.2	14.4	9.8	15.3
	VMAT	27.2	24.3	24.3	24.2	13.9	13.2	11.1	11.2	13.1	12.5	11.7	13.3
	Total	28.1	24.3	23.5	24.1	13.6	16.2	8.1	11.5	12.1	14.0	10.2	14.4
C	3D-CRT	24.2	34.8	22.1	18.9	19.1	14.2	20.6	4.2	17.9	5.6	13.3	5.2
	IMRT	45.0	31.6	10.7	12.7	28.5	21.1	10.5	4.8	5.9	5.3	7.4	16.5
	VMAT	27.4	28.2	20.9	23.5	14.2	13.9	14.3	11.9	9.0	10.3	13.2	13.2
	Total	29.1	35.9	16.5	18.5	17.0	19.3	16.5	8.3	8.2	7.3	11.3	12.1

**Fig. 2:**
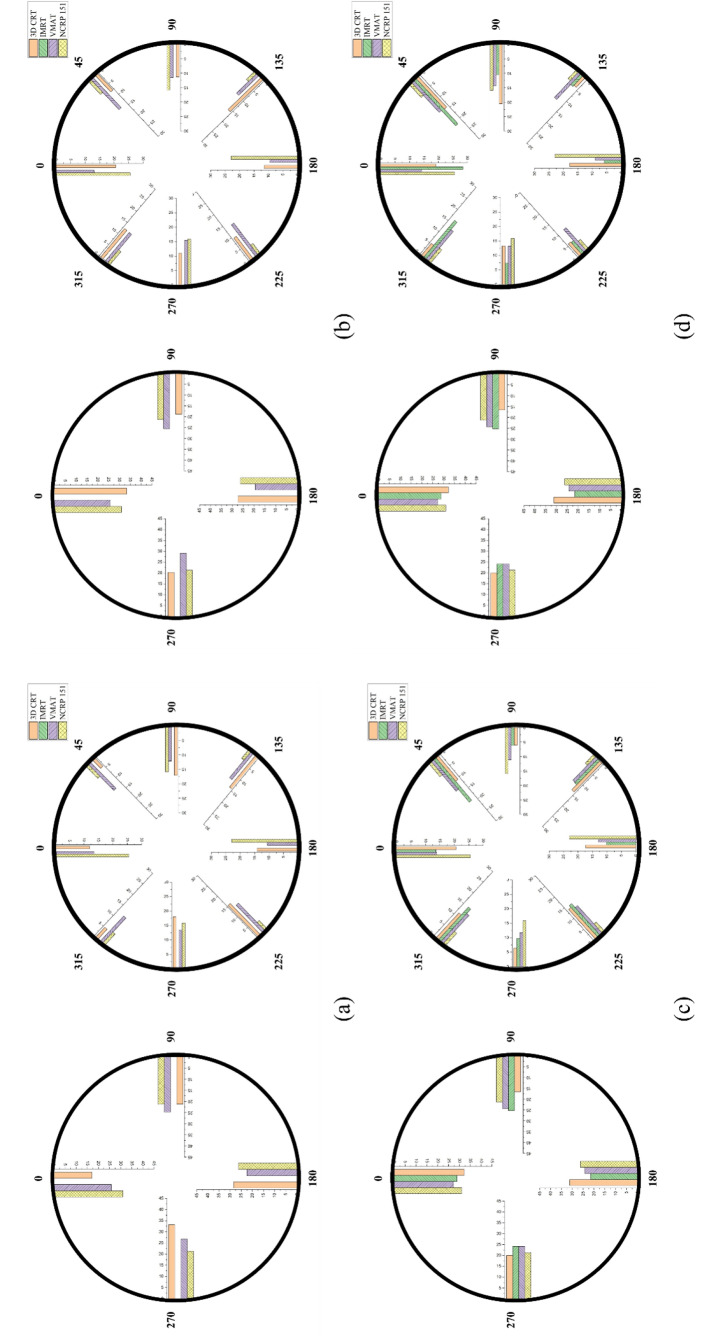
Use factor for the Elekta Infinity LINAC installed at (a) A-1 institute, (b) A-2 institute (c): B institute and (d): C institute: Total gantry angle of 4 bins with 90° intervals, and 8 bins with 45° intervals

The use factor for each LINAC was analyzed according to the treatment technique for 10°, 30°, 45°, and 90° intervals, and the corresponding results are shown in Fig. 2. In general, the use factor is high at a specific angle in the LINAC with a high 3D-CRT ratio compared with that with a high VMAT ratio. IMRT and VMAT show relatively even distributions.

### Use factors for different treatment sites

**Fig. 3:**
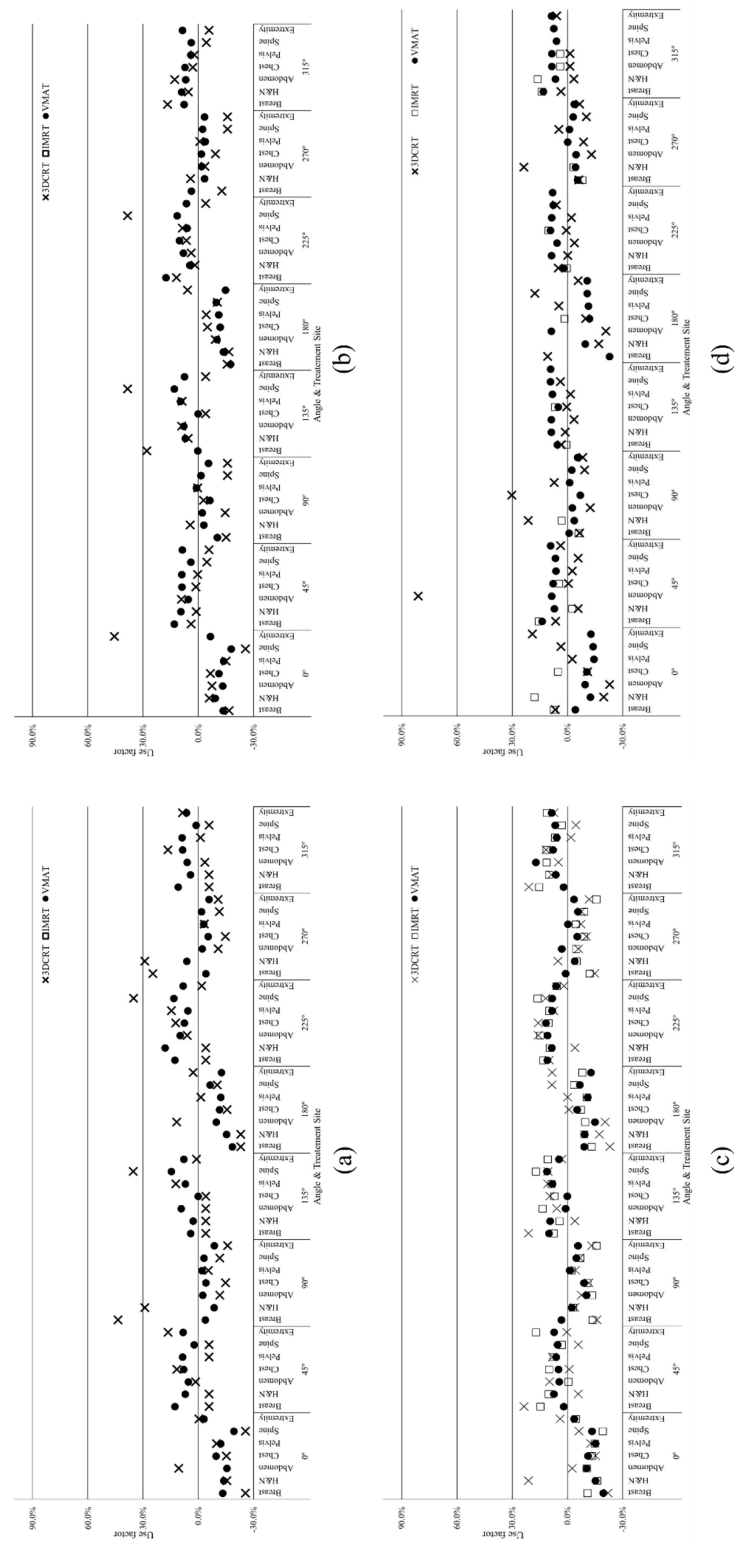
Percent difference between the use factors deduced according to the treatment site and that reported in NCRP 151 for the Elekta Infinity LINAC installed at (a) A-1 institute, (b) A-2 institute (c): B institute and (d): C institute

The results of the percentage differences between the use factor recommended by NCRP 151 and the patient data extracted for each treatment site are displayed in Fig. 3. The differences follow a similar pattern depending on the treatment technique used. We have listed the maximum and minimum differences for each facility and treatment site, along with the corresponding angle, in Table 4.

**Table 4:** Maximum percentage difference between the use factors reported in NCRP 151 and those obtained at each 45° interval according to the treatment technique and treatment area for each treatment room in the institutions

Institution	Treatment technique	Treatment site	Angle	Maximum %difference from NCRP 151
A-1	3D-CRT	Breast	90º	+ 43.6%
		Breast	0º	− 25.6%
	VMAT	H&N*	225º	+ 17.9%
		Spine	45º	− 18.0%
A-2	3D-CRT	Extremity	45º	+ 45.6%
		Spine	45º	− 25.6%
	VMAT	Breast	225º	+ 17.4%
		Spine	45º	− 18.0%
B	3D-CRT	Breast	45º	+ 23.5%
		Breast	180º	− 23.0%
	IMRT	Spine	180º	+ 17.0%
		Spine	45º	− 19.2%
	VMAT	Abdomen	315º	+ 17.1%
		Breast	0º	− 19.6%
C	3D-CRT	Abdomen	45º	+ 81.0%
		Abdomen	0º	− 22.9%
	IMRT	H&N*	0º	+ 17.8%
		Breast	270º	− 8.3%
	VMAT	Breast	45º	+ 13.6%
		Breast	180º	− 22.9%

*H&N: head and neck

## Discussion

The originality of our study lies in the evaluation of use factors according to the treatment technique and treatment site based on the MU weight according to the gantry angle with various intervals. The method of acquiring use factors through DICOM-RT plan files was first attempted by Choi et al. [27], and the use factors were recalculated at 90° intervals. However, in this study, more detailed analysis was performed at intervals of 10°, 30°, 45°, and 90° for each treatment technique and treatment site.

As shown in Table 5, specific recommended use factor values for each angle of the gantry are provided in each report [5, 8, 28–30]. However, it is mentioned that these values can be modified according to the situation in the design example, as they may vary depending on the treatment technique and method. The use factor recalculated through patient data is a factor that considers the gantry angle for the primary beam [5, 8], which can be used to calculate the transmission dose at the point corresponding to the barrier of the primary beam and the maze structure in the treatment room structure.

**Table 5:** A comparison of use factor values for each angle of the gantry presented in the five major reports

Degree	0°	90°	180°	270°
NCRP 49	1.000	0.250	0.250	0.250
NCRP 151	0.310	0.213	0.263	0.213
IAEA 47	0.250	0.250	0.250	0.250
ISO 16,645	0.250	0.250	0.250	0.250
IPEM 75 − 2	0.250	0.250	0.250	0.250

Our study clearly shows that the use factor varies depending on the treatment technique and treatment site. This is evidence confirming NCRP 151’s statement that facilities with a high treatment rate for specific diseases, sites, treatment techniques, and machines may need to evaluate their use factors for radiation safety purposes [2, 12].

It’s important to point out that some machines, such as Tomotherapy, Halcyon, and Cyberknife, use a different method to deliver beams compared to the typical LINAC machines. This means that it’s essential to carry out research at multiple centers to determine the use factor value for these machines [1, 2, 12]. Our research highlights the significance of maintaining radiation safety in healthcare facilities, particularly those that employ radiation therapy for cancer treatment. To ensure the safe and effective use of radiation therapy in the clinical setting, further research and evaluation are necessary.

The use factor recommended by NCRP 151, currently the most referenced, and that calculated from actual patient data in our study show a large difference of 30% or more at most in case of 3D-CRT. In case of VMAT, the difference is not as significant as that in case of 3D-CRT. However, differences are observed depending on the angle. According to other reported studies, the use factor of a stereotactic LINAC that is 100% used for VMAT or dynamic conformal arc therapy should be 0.08, which is a reasonable value, instead of 0.25 recommended by NCRP 151 [26]. Further, comparing the use factors for IMRT and non-IMRT-based treatment of prostate cancer, it is found that at 0°, 75°, 135°, 225°, and 325°, the contribution of IMRT to the output exceeds that of the non-IMRT treatment [31].

According to a different study, use factors are highly dependent on treatment sites [1, 11]. However, even in the same treatment site, the MU delivery weight at a specific angle is different depending on the treatment technique used for each organ; thus, the use factor values may be different. Representatively, an analysis was performed on the results of the use factor used in breast treatment. In the treatment room of institution A and the treatment room of institution C, the use factors for both the VMAT treatment techniques were more than 75%. However, the trend of the use factors used in the two treatment rooms of Institution A was similar; nevertheless, in case of Institution C, the use factor at 0° was approximately twice that of Institution A. Institution B, which mainly uses IMRT treatment, features a high value at 0° and the value at approximately 6° is higher than that at 90°, 180°, and 270°. Although the use factor approaches 0.25 when multiple modalities and techniques are combined, paraspinal stereotactic body radiation therapy (SBRT) features a larger use factor in the ceiling direction [12]. However, as a result of the use factor according to the SBRT treatment technique in Institution B, the value of the use factor facing the floor was the highest at 34.9% and 18% higher than the use factor facing the ceiling. Table 6 shows the results of the recalculated use factor in the previous study and our study, and the percentage difference between the recalculated use factor and the NCRP 151 value. The treatment room with the most difference from the NCRP 151 value is Vault 4 of Choi 2022, showing a difference of 33.8% at 0°. In Choi 2022, Vault 4 explained that the rate of 3D-CRT was higher than other treatment rooms, these results show that even if the treatment area is the same and a similar treatment technique is used, the use factors should be recalculated for each institution.

**Table 6:** In the previous study and our study, the result of the recalculated use factor for each treatment room and the result of the percentage difference with the NCRP 151 value

		User factor by treatment room				Percentage difference from NCRP 151			
		0°	90°	180°	270°	0°	90°	180°	270°
Saleh, 2017	Vault #1	28.4%	23.0%	26.0%	22.6%	-2.6%	1.7%	-0.3%	1.3%
	Vault #2	26.1%	24.7%	25.1%	24.1%	-4.9%	3.4%	-1.2%	2.8%
	Vault #3	25.8%	27.7%	21.9%	24.6%	-5.2%	6.4%	-4.4%	3.3%
	Vault #4	24.1%	12.4%	18.6%	45.0%	-6.9%	-8.9%	-7.7%	23.7%
	Vault #5	15.0%	26.8%	32.7%	25.4%	-16.0%	5.5%	6.4%	4.1%
	Vault #6	11.6%	39.0%	10.6%	38.8%	-19.4%	17.7%	-15.7%	17.5%
	Vault #7	33.2%	20.9%	24.4%	21.5%	2.2%	-0.4%	-1.9%	0.2%
	Vault #8	37.4%	20.2%	22.7%	19.7%	6.4%	-1.1%	-3.6%	-1.6%
	Vault #9	30.4%	29.1%	23.8%	16.7%	-0.6%	7.8%	-2.5%	-4.6%
	Vault #10	32.7%	21.4%	23.6%	22.3%	1.7%	0.1	-2.7%	1.0%
Choi, 2022	Vault #1	26.5%	22.8%	24.0%	26.8%	-4.5%	1.5%	-2.3%	5.5%
	Vault #2	30.8%	22.9%	22.2%	24.1%	-0.2%	1.6%	-4.1%	2.8%
	Vault #3	64.8%	10.6%	10.3%	14.3%	33.8%	-10.7%	-16.0%	-7.0%
	Vault #4	26.5%	21.8%	17.5%	34.2%	-4.5%	0.5%	-8.8%	12.9%
	Vault #5	22.8%	19.9%	23.4%	34.0%	-8.2%	-1.4%	-2.9%	12.7%
	Vault #6	34.8%	21.0%	22.7%	21.5%	3.8%	-0.3%	-3.6%	0.2%
	Vault #7	41.0%	22.7%	16.0%	20.3%	10.0%	1.4%	-10.3%	-1.0%
Our study	Vault #1	25.1%	24.7%	23.6%	26.6%	-5.9%	3.4%	-2.7%	5.3%
	Vault #2	26.3%	24.8%	20.4%	28.5%	-4.7%	3.5%	-5.9%	7.2%
	Vault #3	28.1%	24.3%	23.5%	24.1%	-2.9%	3.0%	-2.8%	2.8%
	Vault #4	29.1%	35.9%	16.5%	18.5%	-1.9%	14.6%	-9.8%	-2.8%

Another factor that can affect the use factor is the field size. In this study, the field size has not yet been taken into consideration, but there are plans to update the program in the future by retrieving the Multileaf Collimator position information for each control point of each field from the DICOM-RT plan file, and calculating the field size to consider in the use factor calculation.

Subsequently modifying the shielding infrastructure through additional re-evaluation is commonly regarded as a challenging task. Therefore, as mentioned in NCRP 151, considering the purpose of the treatment room at the time of design is recommended. However, it may be difficult to predict the use factor in advance because the shielding facility is designed prior to the onset of treatments. Thus, it is necessary to reevaluate the use factor periodically, when the treatment techniques undergo many changes in each institution, or the number of specific treatment sites is increased. The difference between the result obtained at 90° for Institution C and that reported in NCRP was 14.6, which is the largest difference. If the shielding evaluation is performed using the NCRP report value without recalculating the use factor, the agency can underestimate the transmitted dose at a specific point by up to 14.6%. In a treatment room with many specific treatment techniques and treatment sites, dividing the treated patients into different treatment rooms can be an effective alternative.

The use factor may vary depending on the treatment institution or room. Some institutions may use the use factor mentioned in NCRP 151 or other reports as is, while others may recalculate the use factor using actual patient data. We recommend that readers recalculate the use factor using actual patient data because treatment techniques or treatment sites may have changed over time. Some institutions may use the use factor mentioned in NCRP-151 or other reports as is, while others may recalculate the use factor using actual patient data. Since use factor will depend on TPS optimiser, planning practices, treatment site, it is recommended for each site to perform their own analyses based on DICOM files and using the tool. We will provide an online use factor calculation program developed in our study (https://drive.google.com/drive/folders/1xnfjk0uINRZLvyPaTixqPL1k0tvqiTaI?usp=share_link). Using this program, the use factor for each institution can be easily and automatically calculated by inputting the patient’s DICOM-RT plan files and information on shielding facilities. Since each institution’s situation is different, we cannot provide uniform recommendations, but we believe that it will be possible to determine whether using an average in recalculating the use factor by using actual patient data by year, month, and week. Although we may seem to be making somewhat open proposals at present, we expect our program to assist us in making better judgments. In addition, the program can offer a service to determine appropriate reference use factors for newly opened facilities as multi-institutional studies progress and large-scale data accumulates. For the time being, it is possible to use the use factor analyzed from patient data of institutions performing the most similar type of treatment. Similar to previous studies that evaluated the conservatism in the methodology of international protocols and guidelines for the shielding design of linear accelerator facilities [32], the proposed tool in this study can be useful for performing conservative evaluations in treatment rooms where excessive treatments are often performed at specific gantry angles.

The results obtained in this study do not reflect data from many institutions, diverse techniques for total body irradiation, total skin electron beam therapy, SRS, and the quality assurance output. In addition, in the future, analysis of results will be required for new forms of equipment, such as magnetic resonance (MR) LINAC, and the consideration of field size. However, the findings of this study are crucial because it shows the necessity to reevaluate the use factor when the treatment techniques or the number of specific treatment sites change and can be used to analyze the trend of use factor to the most widely used treatment techniques in recent times.

## Conclusion

In our study, we developed a program that automatically calculates use factor from DICOM-RT plan files, and calculated the use factors for four institutions. Based on these results, we showed the need to recalculate use factors for each institution based on patient data. Additionally, we provided a convenient tool for calculating use factors at each institution by making the developed program available online.

## Electronic supplementary material

Below is the link to the electronic supplementary material.

Supplementary Material 1

## References

[1] Cho YR, Jung H, Lee DH (2018). On the use factor analysis and adequacy evaluation of CyberKnife Shielding Design using Clinical Data. Progress in Medical Physics.

[2] Kaur A, Pawaskar P, Sahani G (2018). Mathematical approach in determining use factor for equipment with rotational dose delivery technique. J Med Phys.

[3] National Committee on Radiation Protection and Measurements (1961) Medical X-ray protection up to three million volts: recommendations of the National Committee on Radiation Protection and Measurements.

[4] Burnett BM (1971). NCRP Report No. 34. Medical X-Ray and Gamma-Ray Protection for Energies up to 10 MeV: structural shielding design and evaluation, National Council on Radiation Protection and measurements.

[5] Protection NCoR Measurements (1976) Structural shielding design and evaluation for medical use of X-rays and Gamma Rays of energies up to 10 MeV: recommendations of the National Council on Radiation Protection and measurements. The Council

[6] Protection NCoR (2004) Structural shielding design for medical x-ray imaging facilities. NCRP

[7] NCRP N (2005) Structural shielding design and evaluation for megavoltage x-and gamma-ray radiotherapy facilities 10.1118/1.2336250 28525064

[8] Tran TQ, Jeong S, Nguyen KNH (2006) NCRP Report 151 structural shielding design and evaluation for megavoltage x-and gamma-ray radiotherapy facilities. 10.1088/0952-4746/26/3/B01 10.1118/1.2336250 28525064

[9] Simpkin DJ (1996). Evaluation of NCRP Report No. 49 assumptions on workloads and use factors in diagnostic radiology facilities. Med Phys.

[10] Biggs PJ (2001) Radiation shielding for Megavoltage Therapy Machines in the Post-NCRP 49 era. AAPM Refresher Course, pp 4–20

[11] Kase K (2008) Shielding of Medical Radiation Facilities-National Council on Radiation Protection and Measurements Reports No. 147 and No. 151

[12] Saleh ZH, Jeong J, Quinn B, Mechalakos J, St Germain J, Dauer LT (2017). Results of a 10-year survey of workload for 10 treatment vaults at a high‐throughput comprehensive cancer center. J Appl Clin Med Phys.

[13] Rigo IR, Cunha APV, dos Santos Emiliozzi CZ, Menegussi G (2021) 11-year workload and barrier analysis for a high-energy linear accelerator. Brazilian J Radiation Sci 9. 10.15392/bjrs.v9i2.1687

[14] Otto K (2008). Volumetric modulated arc therapy: IMRT in a single gantry arc. Med Phys.

[15] Palma D, Vollans E, James K, Nakano S, Moiseenko V, Shaffer R, McKenzie M, Morris J, Otto K (2008). Volumetric modulated arc therapy for delivery of prostate radiotherapy: comparison with intensity-modulated radiotherapy and three-dimensional conformal radiotherapy. Int J Radiat Oncol Biol Phys.

[16] Wolff D, Stieler F, Welzel G, Lorenz F, Abo-Madyan Y, Mai S, Herskind C, Polednik M, Steil V, Wenz F (2009). Volumetric modulated arc therapy (VMAT) vs. serial tomotherapy, step-and-shoot IMRT and 3D-conformal RT for treatment of prostate cancer. Radiother Oncol.

[17] Teoh M, Clark C, Wood K, Whitaker S, Nisbet A (2011). Volumetric modulated arc therapy: a review of current literature and clinical use in practice. Br J Radiol.

[18] Roa DE, Schiffner DC, Zhang J, Dietrich SN, Kuo JV, Wong J, Ramsinghani NS, Al-Ghazi MS (2012). The use of RapidArc volumetric-modulated arc therapy to deliver stereotactic radiosurgery and stereotactic body radiotherapy to intracranial and extracranial targets. Med Dosim.

[19] Haertl PM, Pohl F, Weidner K, Groeger C, Koelbl O, Dobler B (2013). Treatment of left sided breast cancer for a patient with funnel chest: volumetric-modulated arc therapy vs. 3D-CRT and intensity-modulated radiotherapy. Med Dosim.

[20] Liu X, Huang E, Wang Y, He Y, Luo H, Zhong M, Qiu D, Li C, Yang H, He G (2017). Dosimetric comparison of helical tomotherapy, VMAT, fixed-field IMRT and 3D-conformal radiotherapy for stage I-II nasal natural killer T-cell lymphoma. Radiat Oncol.

[21] Mutic S, Low D (1998). Whole-body dose from tomotherapy delivery. Int J Radiat Oncol Biol Phys.

[22] Webb S (2000). Conformal intensity-modulated radiotherapy (IMRT) delivered by robotic linac-conformality versus efficiency of dose delivery. Phys Med Biol.

[23] Rodgers JE (2001). Radiation therapy vault shielding calculational methods when IMRT and TBI procedures contribute. J Appl Clin Med Phys.

[24] Mutic S, Low DA, Klein EE (2001). Room shielding for intensity-modulated radiation therapy treatment facilities. Int J Radiat Oncol Biol Phys.

[25] Afrin KT, Ahmad S (2022). 3D conformal, IMRT and VMAT for the treatment of head and neck cancer: a brief literature review. I Radiother Pract.

[26] Rijken J, Bhat M, Crowe S, Kairn T, Trapp J (2019). Linear accelerator bunker shielding for stereotactic radiotherapy. Phys Med Biol.

[27] Choi DH, Kim DW, Ahn SH, Choi SH, Jang YJ, Kwon NH, Seok JH, Park SH, Ahn WS, Kim JS (2022). Shielding evaluator actual treatment leaf: a program for automatic shielding assessment using patient data. Radiat Phys Chem.

[28] IAEA (2006) Radiation Protection in the Design of Radiotherapy Facilities (Safety Report Series Vol 47). International Atomic Energy Agency, Vienna.

[29] International Organization for Standardization (2016). Radiological protection- medical electron accelerators- requirements and recommendations for shielding design and evaluation.

[30] Horton P, Eaton D (2017). IPEM report 75. Design and shielding of radiotherapy treatment facilities.

[31] Mechalakos JG, Germain JS, Burman CM (2004). Results of a one year survey of output for linear accelerators using IMRT and non-IMRT techniques. J Appl Clin Med Phys.

[32] Rijken J, Bhat M, Crowe S, Trapp J (2019). Conservatism in linear accelerator bunker shielding. Australasian Phys Eng Sci Med.

