# Supplementary material for: Development of open access tool for automatic use factor calculation using DICOM-RT patient data
Source: Phys Eng Sci Med. 2023 Jul 20;46(3):1043–53. doi: 10.1007/s13246-023-01272-1 (PMC10480328; doi:10.1007/s13246-023-01272-1)
Supplement: Supplementary file 1 — Supplementary Material 1 [file 13246_2023_1272_MOESM1_ESM.docx]

**Supplement document**

< Use factor for the 4 treatment rooms: Total gantry angle of 12 bins with 30° intervals.>

| Angle(°) |  | A-1 | | | |  | A-2 | | | |  | B | | | | |  | | C | | | | |
| --- | --- | --- | --- | --- | --- | --- | --- | --- | --- | --- | --- | --- | --- | --- | --- | --- | --- | --- | --- | --- | --- | --- | --- |
|  |  | 3D CRT | IMRT | VMAT | Total |  | 3D CRT | IMRT | VMAT | Total |  | 3D CRT | IMRT | VMAT | Total |  | | 3D CRT | | IMRT | VMAT | Total |  |
| 0 |  | 10.8 | 0.0 | 9.0 | 8.6 |  | 19.3 | 0.0 | 8.7 | 9.1 |  | 18.2 | 8.2 | 9.2 | 7.4 |  | | 18.2 | | 19.6 | 9.4 | 7.6 |  |
| 30 |  | 1.9 | 0.0 | 8.2 | 8.1 |  | 3.3 | 0.0 | 8.3 | 8.1 |  | 4.7 | 14.0 | 9.3 | 13.1 |  | | 3.4 | | 15.1 | 9.4 | 5.3 |  |
| 60 |  | 3.7 | 0.0 | 8.4 | 7.4 |  | 5.3 | 0.0 | 10.3 | 10.9 |  | 7.4 | 7.8 | 8.3 | 8.4 |  | | 11.8 | | 11.6 | 9.3 | 4.8 |  |
| 90 |  | 16.0 | 0.0 | 8.3 | 8.6 |  | 10.0 | 0.0 | 7.3 | 6.3 |  | 4.7 | 4.5 | 7.3 | 6.3 |  | | 20.1 | | 6.3 | 9.6 | 49.7 |  |
| 120 |  | 1.8 | 0.0 | 8.3 | 8.1 |  | 8.1 | 0.0 | 7.0 | 4.8 |  | 5.7 | 6.3 | 6.7 | 7.4 |  | | 0.8 | | 5.2 | 9.0 | 3.5 |  |
| 150 |  | 12.7 | 0.0 | 7.8 | 7.9 |  | 8.6 | 0.0 | 6.3 | 5.0 |  | 9.8 | 6.9 | 8.4 | 8.8 |  | | 3.9 | | 2.4 | 6.9 | 4.7 |  |
| 180 |  | 13.6 | 0.0 | 7.2 | 8.6 |  | 11.2 | 0.0 | 6.1 | 6.4 |  | 15.8 | 7.7 | 8.6 | 8.9 |  | | 17.1 | | 4.5 | 5.9 | 9.5 |  |
| 210 |  | 4.1 | 0.0 | 8.3 | 9.2 |  | 2.2 | 0.0 | 8.3 | 8.8 |  | 5.7 | 6.9 | 9.1 | 6.5 |  | | 3.3 | | 3.7 | 6.2 | 3.0 |  |
| 240 |  | 12.4 | 0.0 | 9.2 | 8.3 |  | 8.0 | 0.0 | 10.2 | 11.6 |  | 9.2 | 11.5 | 7.6 | 9.6 |  | | 2.9 | | 3.0 | 7.7 | 2.1 |  |
| 270 |  | 17.1 | 0.0 | 8.8 | 8.9 |  | 9.3 | 0.0 | 10.4 | 12.7 |  | 5.3 | 5.8 | 7.9 | 5.5 |  | | 12.2 | | 5.4 | 9.0 | 3.4 |  |
| 300 |  | 1.8 | 0.0 | 7.9 | 8.4 |  | 8.2 | 0.0 | 8.7 | 8.3 |  | 5.1 | 5.4 | 8.6 | 5.6 |  | | 3.3 | | 8.5 | 8.6 | 2.8 |  |
| 330 |  | 4.0 | 0.0 | 8.7 | 7.8 |  | 6.6 | 0.0 | 8.4 | 8.0 |  | 8.6 | 14.8 | 9.1 | 12.7 |  | | 3.0 | | 14.6 | 9.0 | 3.5 |  |

< Use factor for the 4 treatment rooms: Total gantry angle of 12 bins with 30° intervals.>

| Angle(°) |  | A-1 | | | |  | A-2 | | | |  | B | | | |  | C | | | |
| --- | --- | --- | --- | --- | --- | --- | --- | --- | --- | --- | --- | --- | --- | --- | --- | --- | --- | --- | --- | --- |
|  |  | 3D CRT | IMRT | VMAT | Total |  | 3D CRT | IMRT | VMAT | Total |  | 3D CRT | IMRT | VMAT | Total |  | 3D CRT | IMRT | VMAT | Total |
| 0 |  | 8.4 | 0.0 | 3.1 | 2.8 |  | 17.8 | 0.0 | 2.9 | 3.7 |  | 16.9 | 4.0 | 2.9 | 2.8 |  | 16.9 | 10.4 | 3.1 | 4.7 |
| 10 |  | 1.1 | 0.0 | 2.9 | 3.0 |  | 0.1 | 0.0 | 2.9 | 2.5 |  | 0.3 | 2.1 | 3.3 | 2.8 |  | 0.2 | 3.4 | 3.3 | 2.3 |
| 20 |  | 0.0 | 0.0 | 2.6 | 2.5 |  | 1.2 | 0.0 | 2.7 | 2.5 |  | 1.7 | 2.8 | 3.1 | 2.5 |  | 1.5 | 7.7 | 3.2 | 1.2 |
| 30 |  | 0.3 | 0.0 | 2.8 | 2.8 |  | 1.3 | 0.0 | 2.8 | 2.9 |  | 1.8 | 8.3 | 3.1 | 8.0 |  | 1.1 | 1.9 | 3.1 | 1.6 |
| 40 |  | 2.1 | 0.0 | 2.7 | 2.7 |  | 1.5 | 0.0 | 2.9 | 2.8 |  | 1.9 | 3.8 | 2.9 | 2.4 |  | 1.5 | 7.5 | 2.9 | 2.7 |
| 50 |  | 0.2 | 0.0 | 2.6 | 2.1 |  | 2.7 | 0.0 | 3.3 | 3.8 |  | 3.0 | 2.5 | 2.8 | 3.4 |  | 9.6 | 1.1 | 3.2 | 1.3 |
| 60 |  | 2.1 | 0.0 | 3.1 | 2.7 |  | 1.6 | 0.0 | 4.0 | 4.6 |  | 3.4 | 3.2 | 2.7 | 3.4 |  | 1.3 | 6.4 | 3.1 | 1.6 |
| 70 |  | 0.7 | 0.0 | 2.8 | 2.7 |  | 0.5 | 0.0 | 2.9 | 2.1 |  | 0.2 | 0.3 | 2.5 | 0.8 |  | 0.3 | 0.9 | 3.0 | 1.2 |
| 80 |  | 2.5 | 0.0 | 2.4 | 2.7 |  | 1.4 | 0.0 | 2.1 | 2.1 |  | 0.9 | 3.1 | 2.4 | 3.4 |  | 0.5 | 3.5 | 3.1 | 1.4 |
| 90 |  | 12.9 | 0.0 | 3.0 | 3.1 |  | 7.9 | 0.0 | 2.4 | 2.4 |  | 3.4 | 0.9 | 2.4 | 2.0 |  | 19.4 | 0.3 | 3.3 | 47.0 |
| 100 |  | 0.5 | 0.0 | 2.9 | 3.0 |  | 0.9 | 0.0 | 2.5 | 1.6 |  | 0.6 | 0.8 | 2.4 | 1.0 |  | 0.0 | 4.2 | 3.3 | 1.4 |
| 110 |  | 0.0 | 0.0 | 2.7 | 2.5 |  | 0.6 | 0.0 | 2.3 | 1.6 |  | 0.7 | 1.4 | 2.3 | 2.1 |  | 0.1 | 0.3 | 3.1 | 1.3 |
| 120 |  | 0.2 | 0.0 | 2.8 | 2.8 |  | 3.4 | 0.0 | 2.3 | 1.6 |  | 1.5 | 3.5 | 1.9 | 2.8 |  | 0.1 | 2.6 | 2.9 | 1.0 |
| 130 |  | 2.7 | 0.0 | 2.8 | 2.7 |  | 7.2 | 0.0 | 2.2 | 1.7 |  | 6.2 | 2.1 | 2.5 | 4.0 |  | 1.1 | 0.3 | 2.6 | 1.1 |
| 140 |  | 11.4 | 0.0 | 2.5 | 2.5 |  | 4.4 | 0.0 | 2.0 | 1.6 |  | 3.3 | 3.3 | 2.9 | 2.2 |  | 1.7 | 1.5 | 2.5 | 1.6 |
| 150 |  | 0.0 | 0.0 | 2.6 | 2.6 |  | 0.4 | 0.0 | 2.1 | 1.7 |  | 3.0 | 2.0 | 2.8 | 3.9 |  | 1.6 | 0.1 | 2.1 | 1.4 |
| 160 |  | 0.0 | 0.0 | 2.6 | 2.8 |  | 0.5 | 0.0 | 2.1 | 1.6 |  | 0.7 | 1.2 | 2.9 | 1.5 |  | 0.2 | 1.3 | 2.1 | 2.3 |
| 170 |  | 0.0 | 0.0 | 2.3 | 3.0 |  | 0.5 | 0.0 | 1.9 | 2.0 |  | 0.4 | 2.0 | 3.1 | 2.0 |  | 0.8 | 1.6 | 2.1 | 1.4 |
| 180 |  | 13.0 | 0.0 | 2.2 | 2.8 |  | 10.2 | 0.0 | 1.8 | 2.2 |  | 14.8 | 4.6 | 2.5 | 5.1 |  | 16.2 | 1.4 | 1.7 | 6.7 |
| 190 |  | 1.2 | 0.0 | 2.8 | 2.9 |  | 0.3 | 0.0 | 2.6 | 2.9 |  | 0.5 | 1.1 | 3.1 | 2.3 |  | 0.0 | 1.6 | 2.1 | 0.6 |
| 200 |  | 0.0 | 0.0 | 2.5 | 3.0 |  | 0.0 | 0.0 | 2.5 | 2.7 |  | 0.9 | 3.4 | 3.0 | 2.2 |  | 0.8 | 0.0 | 2.0 | 0.6 |
| 210 |  | 0.0 | 0.0 | 2.9 | 3.3 |  | 0.2 | 0.0 | 3.0 | 3.2 |  | 3.0 | 1.6 | 2.9 | 2.3 |  | 1.6 | 2.5 | 2.0 | 1.5 |
| 220 |  | 7.0 | 0.0 | 2.9 | 2.9 |  | 3.6 | 0.0 | 3.0 | 3.0 |  | 3.2 | 2.6 | 3.1 | 1.6 |  | 1.7 | 0.8 | 2.3 | 1.1 |
| 230 |  | 7.8 | 0.0 | 2.9 | 2.6 | 0.0 | 3.9 | 0.0 | 3.1 | 3.2 | 0.0 | 4.9 | 5.8 | 2.7 | 5.2 | 0.0 | 0.9 | 0.4 | 2.4 | 0.6 |
| 240 |  | 0.5 | 0.0 | 3.2 | 2.8 | 0.0 | 2.2 | 0.0 | 3.6 | 4.4 | 0.0 | 2.6 | 2.3 | 2.1 | 2.6 | 0.0 | 1.0 | 2.0 | 2.7 | 0.5 |
| 250 |  | 1.2 | 0.0 | 3.2 | 2.8 | 0.0 | 0.1 | 0.0 | 4.0 | 4.9 | 0.0 | 0.3 | 4.1 | 2.6 | 2.1 | 0.0 | 0.2 | 0.3 | 2.9 | 0.8 |
| 260 |  | 0.5 | 0.0 | 2.8 | 2.8 |  | 0.5 | 0.0 | 3.5 | 4.4 |  | 1.9 | 1.7 | 2.6 | 2.4 |  | 0.4 | 3.5 | 3.1 | 0.9 |
| 270 |  | 15.9 | 0.0 | 3.0 | 3.2 |  | 7.7 | 0.0 | 3.3 | 4.2 |  | 3.3 | 0.8 | 2.7 | 0.9 |  | 11.3 | 0.4 | 3.0 | 1.8 |
| 280 |  | 0.3 | 0.0 | 2.7 | 3.0 |  | 2.0 | 0.0 | 3.0 | 3.3 |  | 0.0 | 2.5 | 2.6 | 2.3 |  | 0.9 | 2.5 | 2.8 | 0.7 |
| 290 |  | 0.2 | 0.0 | 2.5 | 2.9 |  | 0.2 | 0.0 | 2.8 | 2.7 |  | 1.0 | 0.5 | 2.8 | 1.4 |  | 1.0 | 0.3 | 2.7 | 0.7 |
| 300 |  | 1.4 | 0.0 | 2.7 | 2.7 |  | 4.1 | 0.0 | 3.0 | 2.7 |  | 2.9 | 2.7 | 3.0 | 1.9 |  | 1.4 | 6.1 | 2.9 | 0.8 |
| 310 |  | 0.0 | 0.0 | 2.9 | 2.5 |  | 5.7 | 0.0 | 2.9 | 2.6 |  | 2.4 | 2.1 | 3.0 | 2.4 |  | 1.0 | 1.7 | 3.1 | 2.1 |
| 320 |  | 3.0 | 0.0 | 2.8 | 2.5 |  | 1.2 | 0.0 | 2.6 | 2.5 |  | 2.9 | 2.5 | 3.0 | 1.6 |  | 1.2 | 6.0 | 2.8 | 0.8 |
| 330 |  | 0.6 | 0.0 | 3.0 | 2.7 |  | 2.1 | 0.0 | 2.9 | 2.9 |  | 3.5 | 10.3 | 3.1 | 8.9 |  | 1.2 | 3.3 | 3.1 | 0.8 |
| 340 |  | 0.9 | 0.0 | 3.0 | 2.9 |  | 0.9 | 0.0 | 2.9 | 2.8 |  | 2.0 | 2.2 | 3.0 | 2.1 |  | 0.2 | 9.0 | 3.1 | 1.7 |
| 350 |  | 1.4 | 0.0 | 2.9 | 2.8 |  | 1.0 | 0.0 | 2.9 | 2.8 |  | 0.2 | 2.0 | 3.2 | 2.2 |  | 1.1 | 2.9 | 3.2 | 0.9 |
